# Which factors decided general practitioners’ choice of hospital on behalf of their patients in an area with free choice of public hospital? A questionnaire study

**DOI:** 10.1186/1472-6963-12-126

**Published:** 2012-05-25

**Authors:** Hans O Birk, Lars O Henriksen

**Affiliations:** 1 Region Zealand, Quality and Development, Alléen 15, 4180, Sorø, Denmark; 2 University of Copenhagen, Department of Public Health, Øster Farimagsgade 5, P.O. Box 2099, 1099, København K, Denmark

## Abstract

**Background:**

Parts of New Public Management-reforms of the public sector depend on introduction of market-like mechanisms to manage the sector, like free choice of hospital. However, patients may delegate the choice of hospital to agents like general practitioners (GPs). We have investigated which factors Danish GPs reported as decisive for their choice of hospital on behalf of patients, and their utilisation of formal and informal data sources when they chose a hospital on behalf of patients.

**Methods:**

Retrospective questionnaire study of all of the 474 GPs practising in three counties which constituted a single uptake area. Patients were free to choose a hospital in another county in the country. The GPs were asked about responsibility for choice of the latest three patients referred by the GP to hospital; which of 16 factors influenced the choice of hospital; which of 15 sources of information about clinical quality at various hospitals/departments were considered relevant, and how often were six sources of information about waiting time utilised.

**Results:**

Fifty-one percent (240 GPs) filled in and returned the questionnaire. One hundred and eighty-three GPs (76%) reported that they perceived that they chose the hospital on behalf of the latest referred patient. Short distance to hospital was the most common reason for choice of hospital.

The most frequently used source of information about quality at hospital departments was anecdotal reports from patients referred previously, and the most important source of information about waiting time was the hospitals’ letters of confirmation of referrals.

**Conclusions:**

In an area with free choice of public hospital most GPs perceived that they chose the hospital on behalf of patients. Short distance to hospital was the factor which most often decided the GPs’ choice of hospital on behalf of patients. GPs attached little weight to official information on quality and service (waiting time) at hospitals or departments, focusing instead on informal sources like feedback from patients and colleagues and their experience with cooperation with the department or hospital.

## Background

A common trait in public sector governance reforms in the Nordic countries in the latest two decades is a gradual development from collective systems towards an individual-based democracy model [1], where individual citizens are viewed as autonomous consumers rather than clients [2] and are expected to set priorities and allocate resources by utilising consumers’ rights [3] to choose treatment, appointment times and/or providers [4]. In general the interest in introducing choice is based on two fundamental arguments [5,6]: an ideological viewpoint, which views an opportunity for citizens to choose a supplier as an objective in itself, as it strengthens personal freedom [7,8], and an instrumental viewpoint, which emphasizes that the public sector can improve its effectiveness, quality, equity in access to care and responsiveness by introducing or strengthening choice, e.g. an opportunity for patients to choose a health care provider [2,4]. In combination with activity-based payments, where “the money follows the patient” [9-13] choice is assumed to constitute a self-correcting allocation mechanism, which resembles the market mechanism in competitive markets [9,13], as providers who provide less than optimal care may be “punished” by customers through exit [14]. Thereby, ideally, individual actors’ utility maximisation on the demand side as well as the supply side results in an optimal resource allocation and production in society [4,15,16].

One “model of patient choice as a governance tool” builds on several preconditions [4], including ten preconditions concerning patients’ and general practitioners’ (GPs’) knowledge, assumptions and behaviour:

· Patients are aware of their ability to choose

· Patients want to choose and think choice is important

· Patients are offered choice of providers

· Quality is the primary discriminator in patients’ choice of which provider to attend

· Patients have access to relevant and appropriate information on quality and are able to interpret the data

· GPs believe that choice is important to patients

· GPs offer choice to all patients needing a referral

· GPs involve patients in decision-making

· GPs have access to information about the quality of providers and convey this information to patients

· GPs have time and resources to support patients to make an informed choice [4]

However, while choice in theory could be a driver for improving quality and service in health care these preconditions from neoclassical microeconomic theory are only fulfilled to some degree [17]. For example patients appear to utilize information only if there is a single outcome of major importance and the data is easy to understand [18], many patients being insufficiently informed to utilize data for choice resulting in market failure [19,20] and thereby reducing the potentially positive impact of choice on quality and service [17]. Patients may be reluctant to take responsibility for choosing the hospital in order to avoid regretting their choice [21-23], preferring to enter into a principal-agent-relationship with an intermediary. In an ideal principal-agent-relationship the agent (i.e. the GP) makes the decision, which the principal (i.e. the patient) would have made, if the principal had had the same information as the agent about the expected effect of various interventions and the quality of individual providers’ services. English, Dutch and Danish surveys have shown that GPs choose the hospital on behalf of a major share of patients, even when patients’ awareness of their right to choose is high [24-26], and the GP is the most important source of information for a major share of the patients who choose the hospital by themselves [24,26-28], patients being even more sensitive to GPs’ warnings against specific hospitals than to their recommendations of specific hospitals [28]. In 2004 87% of all Danish elective in-patients were aware of freedom of choice of hospital, and 42% of all elective patients chose the hospital by themselves (of which 30% attributed major influence to the GP on their choice). The GPs chose the hospital on behalf of 58% of patients, who were not aware of choice or delegated the choice to the GP [26].

An English study found varying support for choice among GPs, and choice has not changed the GPs’ behaviour towards more emphasis on advice on choice [29]. GPs may be reluctant to provide advice to patients and promote utilisation of choice, because this task competes with other tasks; they do not consider this task a part of their job [4,30]; consider this task too time-consuming [4]; distrust data published by the providers [29], or want to avoid being blamed by patients for presenting faulty data [23]. Therefore GPs may choose the hospital on behalf of patients rather than provide advice to the patients, thereby reducing patients’ influence on the governance-effect of patient choice.

We investigated:

· Whether GPs considered the patients or themselves to be responsible for choice of hospital?

· Which factors decided GPs’ choice on behalf of patients?

· Which formal and informal data sources were utilised by GPs in choice on behalf of patients?

The study was performed in a setting, where patients and GPs had one decade’s experience with free choice of hospital; where patients’ awareness of choice was high, and where the share of patients for whom GPs chose the hospital had been stable at a high level for several years.

Hospital care was provided free at the point of delivery by a universal, tax-financed, public health care system. In the study period the citizen’s home county was responsible for provision of health care performed by GPs, specialists, the county’s hospitals, or other counties’ hospitals (by patients utilising choice or by patients referred to hospitals performing highly specialised interventions). Each citizen had to register with a local GP, who was responsible for basic examinations and treatments. GPs, acting as gatekeepers, decided whether a patient should be referred to hospital for elective care, and could refer a patient to any public Danish hospital or specialist for specialised services. In case of emergency, patients had direct access to hospital but could not choose the hospital by themselves. GPs were self-employed and responsible for their own facilities and never performed their tasks in a hospital, unlike in the US [31].

The GPs were paid by the counties in proportion to 1) the number of patients registered with them (capitation, approx. 1/3 of GPs’ income), and 2) the number of services they provided to their patients (fee-for-service, approx. 2/3 of GPs’ income). The payments to GPs were independent of the number of referrals and the choice of hospital [31].

Elective patients could choose the hospital during the visit to the GP or after the visit to the GP but before going to the hospital. If more than one hospitalisation was indicated, the patients could choose another hospital at any time before the last hospitalisation. If the patient did not make the choice by themselves, the GP chose the hospital, by filling in a referral form on paper (no computerised facility like the English “Choose and Book” was available). Filling in the form took equally long time whether the patient was referred to a local hospital or a hospital in another Danish county. GPs did not receive any kind of incentive payment for advising patients on choice of hospital [31].

Patients referred to hospital were responsible for transportation arrangements and costs. However a patient was entitled to transportation or a refund of his/her transportation costs by the county, if the patient was a pensioner, lived more than 50 km by road from the nearest hospital which could perform the procedure, or could not utilise public transport for health reasons. If a patient was entitled to a refund of transportation costs due to the distance criterion and chose a more distant hospital, the refund was calculated based on the distance to the closest hospital capable of performing the procedure.

Danish public hospitals were owned and managed by a regional political/administrative level: the counties. The private Danish hospital sector owned less than 1% of Danish hospital beds in the study period. Danish hospitals provided in-patient as well as out-patient care. If a patient was referred to a hospital outside the home county, the home county/region paid a DRG-charge to the county which owned the hospital performing the treatment, thereby creating a financial incentive for the counties to attract patients from the county as well as patients from other counties to the county’s hospitals, but hospitals were not obliged to accept elective patients from other counties. Hospitals’ and hospital departments’ income grew with the production of DRG points up to a certain level. To avoid discrimination against patient groups, hospitals and departments received the same payment for treatment of patients independent of where the patients lived [31].

Elective patients could choose any public hospital, a broad majority in the Danish parliament having introduced “free choice of [public] hospital” in Denmark in 1993 with several parallel objectives in mind including a view on choice as a patients’ right, to level out waiting times, strengthen patients’ influence on the hospital sector, improve hospitals’ treatment results and improve patients’ satisfaction [32]. However, significant limitations on patients’ rights and on hospitals’ financial incentives to accept patients from other uptake areas were introduced due to fear that a more demand-driven health care sector would lead to budget overruns. Gradually the government extended patients’ freedom to choose and strengthened hospitals’ financial incentives to accept patients, thereby creating a common market at the national level for elective, public health care. In 1991, before the introduction of free choice at the national level, three counties in Eastern Denmark independently introduced free choice within their own area (the study area).

The counties and the Ministry of the Interior and Health published waiting time forecasts for common elective treatments at hospital level and results of biannual surveys of patients’ experience with individual hospitals (but not with individual departments). The ministry published data on individual departments’ volume for common surgical interventions as a proxy for quality on the assumption that department volume was associated with experience and thereby clinical quality. Data on other aspects of service or clinical quality at clinics was not published systematically, but some departments and medical societies published data on individual departments’ performance as part of quality development or clinical research.

## Methods

The present study was performed in the three mixed urban/rural counties of Roskilde, Storstrøm and Vestsjælland (801,452 inhabitants on January 1 2004 in total) in Eastern Denmark. The counties in the study area provided hospital treatment at 13 public hospitals evenly distributed within the region. No point in the study area was more than 30 km from the nearest public hospital in a bee line. Each specialty represented in the study area was available at two hospitals or more, except for dermatology and plastic surgery, which were only available at one hospital each. Patients were free to choose treatment, paid by the home county, at hospitals in other Danish counties. Patients in need of care at tertiary hospitals were referred to hospitals outside the study area from the counties’ own hospitals. Each public hospital was obliged to accept any referral from any GP in any of the three counties. Patients who were entitled by law to free travel to the hospital closest to their home were offered free travel to any public hospital in the study area, thereby strengthening patients’ opportunities to utilise their freedom of choice.

The three counties jointly published waiting time-prognoses for common surgical procedures at hospitals within the study area. At first these prognoses were mailed to each GP on paper, later they were published on a website managed by the Danish counties and accessible to the public (http://www.sundhed.dk) and a national website maintained by the Ministry for the Interior and Health (http://www.venteinfo.dk). Departments held regular information meetings for the GPs about the interventions provided at the department and the department’s procedures.

The study group included all of the 483 GPs registered as practising in the study area. The names and addresses of the GPs were found by use of the Danish counties’ website, http://www.sundhed.dk.

The study was performed as a questionnaire study. The retrospective design was chosen to avoid influencing the GPs’ choice behaviour.

The questionnaire was developed after a review of the literature [33-35] and face-to-face discussions with GPs from two of the three counties. The questionnaire was validated by interviews with three GPs: two GPs practising in a large and a small town in the study area, respectively, and one affiliated with the University of Copenhagen’s Section of General Practice. The three GPs were asked whether the questions were unambiguous, and whether the predefined answers were sufficient. The GPs’ interpretation of the questions was compared with the authors’ intentions. Based on the GPs’ responses several open and closed responses were added to the questionnaire, for example hospitals’ confirmation of reception of referrals and clinical reports to the GPs after discharge was included as a source of proxy information on waiting time. The GPs also emphasized the importance for choice of hospital of hospitals’ attitude to the GPs and the cooperation between hospital and GP, and these reasons for choice of hospital were added to the questionnaire.

The final questionnaire included the following questions ( Additional file 1):

· For the GP: gender and year of birth.

· For each of the latest three somatic patients referred to hospital (department or out-patient clinic) by the GP for treatment: gender, year of birth, and the specialty the patient was referred to. Who chose the hospital in the GP’s opinion (the patient, the GP or the patient’s relatives)? Which of 16 factors influenced the choice of hospital strongly in the GP’s opinion (see Table 1 for the list of factors; the GPs could tick off as many factors as they found relevant)? How many factors influenced the choice? The GP could add comments on responsibility for choice and on the 16 factors.

· For the GP: which of 15 sources of information on quality at department level did the GP in general consider most relevant (see Table 2 for the list of sources of information; the GPs could tick off as many factors as they found relevant)? How often did the GP use six specified sources of information on expected waiting time at hospital departments (routinely (4 points); often (3 points); rarely (2 points); not at all (1 point); see Table 3 for a list of the sources of information)? The GP could add comments on the sources of information on quality as well as on waiting time.

**Table 1:** Factors deciding 216 GPs’ choice of hospital on behalf of their most recent patient referred to hospital

**Decisive factor**	**Number of GPs (%)**
The department was the closest to the patient’s home	187 (85%)
The department takes the GP’s referrals seriously	60 (27%)
Excellent cooperation between GP and department	56 (26%)
Comments from patients referred to the department by the GP	54 (25%)
The patient had been treated at the hospital before	47 (21%)
The patient had been treated at the department before	44 (20%)
The hospital takes the GP’s referrals seriously	44 (20%)
Comments from patients referred to the hospital by the GP	41 (19%)
The department provides detailed clinical reports	39 (18%)
The hospital provides detailed clinical reports	33 (15%)
The department sends clinical reports soon after discharge	24 (11%)
The hospital sends clinical reports soon after discharge	20 (9%)
The GP´s experience as a trainee at the hospital	11 (5%)
The GP´s experience as a trainee at the department	15 (7%)
Waiting time was shorter than at other departments	11 (5%)
Total	686
Number of referrals	216
Number of factors quoted/referrals	3.2

**Table 2:** General practitioners’ sources of information on quality at hospital departments (number and share of 240 GPs)

**Source of information**	**Number and share of respondents**		
	**All GPs**	**Female GPs**	**Male GPs**
Patients’ comments on the department	160 (66%)	51 (68%)	109 (66%)
Other GPs’ comments on the department	131 (54%)	42 (56%)	89 (54%)
Patients’ comments on the hospital	128 (53%)	40 (53%)	88 (53%)
The GP’s acquaintance with hospital personnel	91 (38%)	23 (31%)	68 (41%)
Other GPs’ comments on the hospital	89 (37%)	31 (41%)	58 (35%)
Official information from the department	85 (35%)	39 (52%)****	46 (28%)****
Clinical reports from different departments	74 (31%)	23 (31%)	51 (31%)
Information meetings in hospital departments	74 (31%)	23 (31%)	51 (31%)
Official information from the hospital	71 (30%)	32 (42%)**	39 (23%)**
The GP’s trainee experience at the hospital	54 (22%)	21 (28%)	33 (20%)
The GP’s trainee experience at the department	47 (20%)	16 (21%)	31 (19%)
The hospital’s description of its quality standards	7 (3%)	4 (5%)	3 (2%)
The department’s description of its quality standards	6 (3%)	3 (4%)	3 (2%)
Media reports about the hospital	5 (2%)	3 (4%)	2 (1%)
Media reports about the department	4 (2%)	2 (3%)	2 (1%)
Number of sources ticked off by the GPs	1,027	354	673
Number of respondents	240	75	165
Sources/respondent	4.3	4.7	4.1

**: *p* < 0.01. ****: *p* < 0.001.

**Table 3:** General practitioners’ use of various sources of information on expected waiting time at hospitals (n = 241)

**Source of information**	**Routinely (4)**	**Often (3)**	**Rarely (2)**	**Not at all (1)**	**Average (1–4)**
Confirmations/clinical reports	45 (19%)	84 (35%)	67 (28%)	44 (18%)	2,5
The counties’ prognoses (paper)	28 (12%)	64 (27%)	73 (30%)	75 (31%)	2,2
Calls to the departments	2 (1%)	29 (12%)	141 (59%)	68 (28%)	1,9
The counties’ prognoses (web)	12 (5%)	36 (15%)	78 (32%)	114 (48%)	1,8
http://www.venteinfo.dk	7 (3%)	31 (13%)	84 (35%)	119 (49%)	1,7
http://www.sundhed.dk	2 (1%)	9 (4%)	63 (26%)	166 (69%)	1,4

A patient and a GP may share the choice of hospital [36], but our objective was to establish which person the GP considered to have the greatest influence on choice, and how they chose the hospital on behalf of patients rather than investigate shared decision-making. Therefore the GPs could not respond that they shared the decision with the patient.

To increase the GPs’ response rate the questionnaire was limited to four A4-pages, the questionnaire was mailed by first-class-post, and a stamped return envelope was enclosed [37]. The questionnaire was mailed to the study group in December 2003. GPs who did not respond within a month received a single reminder. We compared respondents and non-respondents by county, number of years since graduation (available from the Danish MDs’ Association’s “Who’s Who”) and gender (deduced from the GPs’ names).

The following data was recorded: the number of patients where the hospital was chosen by the GP, the patient or the patient’s relatives. For GPs who had chosen the hospital on behalf of one patient or more we recorded the reasons for the choice on behalf of the latest patient referred to hospital, for which the GP reported that he or she made the choice. Each GP was only included once in the study of reasons for choice to avoid mutually dependent observations.

Data was recorded in a database (EPIINFO Version 3.2.2. April 14, 2004). Respondents were compared with the study population by univariate analyses of gender, county (chi²) and number of years since graduation (t-test). This analysis was repeated for GPs who had chosen the hospital on behalf of at least one patient.

For GPs who had chosen the hospital on behalf of one or more patients we recorded the GP’s reasons for choice on behalf of the most recent patient to minimize recall bias. GPs’ reasons for choice were compared by univariate analysis for gender (chi²) and years since graduation (t-test), and by logistic multiple regression analysis with the GPs’ gender and years since graduation as the independent variables. We tested for correlation between the number of factors for choice and the GPs’ gender and years since graduation by use of a multiple regression analysis. The GPs’ use of information sources on quality and expected waiting time at various hospitals were compared by univariate analyses for association with gender (chi²) or years since graduation (t-test) and by multiple logistic regression analysis with gender and years since graduation as the independent variables.

The study was performed in accordance with the Helsinki Declaration. According to section eight in the Danish Act on a Biomedical Ethics Committee System and the Processing of Biomedical Research Projects questionnaire studies were not notifiable to the Danish research ethics committee system, if they did not include biological material [38].

## Results

A questionnaire was sent to the 483 GPs listed in the database. Nine of the registered GPs in the database represented data errors, reducing the study population to 474 GPs. Two hundred and forty GPs (51%) returned a filled-in questionnaire. Male and female GPs’ response rates were 50% and 54%, respectively. Respondents did not differ significantly from the study population with regard to county, gender or number of years since graduation.

### Responsibility for choice of hospital

Among the 240 respondents 183 (76%) reported that in their view they chose the hospital on behalf of the latest patient referred to hospital, 35 (15%) reported that the patient made the choice, two (1%) reported that the patients’ relatives made the choice, and 20 (8%) did not state, who chose the hospital or ticked off several categories. Several of these GPs commented that they chose the hospital in cooperation with the patient or that the patient agreed with the GP. One GP commented that he always asked whether the patient wanted to be referred to another hospital than the one proposed by the GP, and another GP reported that he asked whether the patient wanted to be referred to a specific department.

### Reasons for GPs’ choice of hospital

Ninety-two percent of the respondents (220 of 240) reported that they had chosen the hospital on behalf of one patient or more, while 20 GPs attributed all patients’ choice to the patients, their relatives or referral guidelines limiting free choice of hospital for the specific patients (these guidelines did not interfere with patient’ rights to choose a hospital in another county). The 220 respondents did not differ significantly from the study population with regard to county, gender or number of years since graduation. Four GPs did not tick off any reasons for choice of hospital and were excluded from this part of the study. Eighty-seven of the 216 GPs (40%) reported that a single factor decided their choice for the patient, short distance to the hospital being the decisive factor for 75 of the 87 GPs (86%). Ninety-five GPs reported that 2–5 factors were very important for their choice, 25 quoted 6–9 factors, and nine GPs quoted ten or more factors.

Short distances to hospital, the department’s serious consideration of referrals from the GP, and comments from previous patients referred to the department were the most common factors behind GPs’ choice of hospital on behalf of patients (Table 1).

The importance of each factor behind choice of hospital was not associated with the GP’s gender (data not shown), and multiple regression analysis showed no significant association between the number of reasons for choice and the number of years since the GP’s graduation (β = 0.109, *p* = 0.26) or gender (*p* = 0.96). In univariate analysis of each factor and the number of years since graduation, GPs who based their choice on their personal experience with the department as employees were significantly younger (on average 20.1 years since graduation) than GPs who did not (on average 24.2 years since graduation) (*p* < 0.05).

One GP commented that problems associated with transport and rehabilitation after hospitalisation posed greater challenges than waiting time, and therefore patients were only referred out of the county if the quality of care within the county was very bad.

### Sources of information on quality or service

The most frequently used sources of information on quality at hospital departments were reports from patients referred to the department or the hospital by the GP previously (Table 2), and other GPs’ comments on the department. Univariate analysis showed that female GPs were significantly more likely than male GPs to consider official information from departments an important source of information, and younger GPs were significantly more likely than older GPs to quote their experience as trainees at a department (average number of years since graduation 19.2 and 25.1 respectively, *p* < 0.001) or a hospital (average number of years since graduation 21.4 and 24.7 years respectively; *p* < 0.05) or comments from patients previously referred to a department (average number of years since graduation 23.2 and 25.3 years respectively; *p* < 0.05) as important sources of information on quality. Multivariate analyses confirmed that there were statistically significant negative associations between the number of years after the GPs’ graduation and GPs’ utilisation of information from previously referred patients (β = −0.05; *p* < 0.05), and an association between gender and use of official information from the department (odds ratio 0.37; *p* < 0.01) or the hospital (odds ratio 0.35; *p* < 0.01), female GPs being more likely to quote official information as a source of information. Multivariate analysis found no association between GPs’ age or gender and their quoting experience from employment at department or hospital as sources of information on quality (data not shown).

One GP commented pointedly that she spent her scarce time on the patients rather than reading official information about quality at various departments. Several GPs repeated in their response to this question their answers to another question that previous patients’ reports were the most important sources of information on quality, two GPs underscoring that they attributed greater weight to 20 years’ experience with a hospital than to the hospital’s description of its quality, one GP contrasting “action” with “words”.

The hospitals’ letters of confirmation of referrals were the GPs’ most important source of information on waiting times (Table 3). Information available from websites was used less often than information on paper. Univariate analysis found no association between GPs’ age or gender and their utilisation of various sources of information, but multiple, logistical regression analyses showed that male (*p* < 0.01) and younger GPs (*p* < 0.01) were especially likely to use the counties’ waiting time prognoses on the internet.

Eight GPs reported that they often asked patients to call the county’s patient advisor to discuss which department they would like to be referred to, one GP adding that in some cases she recommended accepting a long waiting time if the department was an especially good one. One GP commented that in her opinion it was a task for the hospital to inform the patients about waiting times, while another GP had delegated collecting of and information about data on waiting times to her secretary. Two GPs reported that if patients wanted a shorter waiting time, they asked the patients to look for waiting time data elsewhere. One GP likewise stated that she informed the patient about the right to free choice but asked them to investigate the opportunities on their own. One GP reported that she did not use any official information about expected waiting times, because waiting time prognoses were outdated as early as at the time of publishing. Several GPs described an intention to utilize data on the web in the future, although some GPs found that utilization of data on the internet was a very time consuming and complicated process. One GP found that calling departments likewise was time consuming because it usually took a long time to find somebody who could answer questions on expected waiting time.

## Discussion

### Responsibility for choice of hospital

In the present study the GPs reported that they chose the hospital on behalf of 76% of patients. This result appears to contradict results from national Danish surveys of patients’ experience with hospitals: in 2004 46% of in-patients treated in the study area reported, that they chose the hospital; among elective in-patients 89% were aware before being hospitalised that they were free to choose, and 52% of these patients chose the hospital by themselves [26]. The divergent findings may be interpreted as an indicator of shared decision making. When 30% of patients reported that their GP’s recommendation influenced their own choice of hospital [26], the GPs may have perceived that they chose the hospital on behalf of the patient.

The results indicate that patients choose the hospital to a lesser degree than policy makers (politicians and administrators) want them to do to improve management of the public health care sector by introducing a proxy for the market mechanism. Other studies have found that GPs appear to question whether choice is valuable to patients [29], and whether patients really want to choose the hospital [4]. GPs’ choice behaviour varies by GP [29] and by the patients’ diagnoses [39], English GPs being more likely to offer choice to patients, who are in need of a routine intervention, elective patients, and patients who are relatively healthy [4].

One study distinguished between ‘choice enthusiasts’, ‘choice sceptics’ and ‘choice paternalists’ [29]. The present study did not enable us to divide GPs into such subgroups, but confirming results from other studies [4,30] several GPs expressed reluctance to provide advice to patients, because they did not consider this task a part of their job [30,40]; considered this task too time-consuming for a consultation [4], or distrusted data published by the providers [29] and wanted to forestall blame for presenting faulty data [23]. This behaviour may reflect an attempt to minimize the length of each visit to the GP. However, at a more general level GPs’ behaviour may reflect a ‘logic of care’ rather than a ‘logic of choice’ [41] - GPs making choices based on their professional views on patients’ needs and wants, rather than as agents acting in a market place enabling patients to make informed choices in line with the neoclassical standard model.

### Factors determining GPs’ choice of hospital on behalf of patients

Short distance to hospital was the most important factor behind GPs’ choice of hospital. Numerous other studies of GPs’ actual referral pattern and patients’ choices in structurally different health care systems likewise indicate that short distance strongly influences patients’ and GPs’ choice of hospital [26,33,42]. Studies of GPs’ hypothetical referrals and patients’ hypothetical choices have led to other results with GPs emphasizing the importance of short waiting time and the GP’s impression of quality at the alternative departments [35,43], while patients facing a hypothetical choice emphasized the importance of data on structure quality and attributed little weight to waiting time [44].

Different findings in studies of GPs’ and patients’ choice behaviour may reflect differences between how GPs and patients think they ought to choose the hospital and how they actually make the choice, one study finding significant differences between GPs’ response to hypothetical case stories and their actual referral pattern [45]. Another reason could be international institutional differences with regard to subsidization of transport costs and the length of waiting times.

The small influence of waiting time on choice may be considered to be remarkable, as the media and politicians at the national level consistently focus on waiting times as a major performance measure and challenge, but other studies of choice of hospital likewise found only a small influence of waiting time on choice. Cataract patients generally accepted waiting times of three months and less, while waiting times of six months or more were perceived as too long [46,47]. In a hypothetical study patients reported that for each additional hour of travel time they would, on average, require a reduction of in the waiting time of 2.3 months [48]. The results of these studies and the present study may partly explain why differences between waiting times at hospitals persisted more than a decade after the introduction of free choice of hospital, but they may also reflect, that a minority of patients are treated as elective patients.

In the present study we focused on the influence of GPs’ sources of information about departments/hospitals and factors commonly found to influence the GPs’ choices. However, GPs’ choices on behalf of patients may be influenced by other agendas independent of the individual patient, i.e. GPs may refer patients to a local hospital to contribute to its continuing existence [30].

### GPs’ use of sources of information on quality and service

In the present study GPs were less likely to use official information on quality and waiting time than proxy-measures from informal sources like their own and other GPs’ and patients’ experience with regard to quality and waiting time. This result was consistent with other studies of GPs’ or patients’ utilisation of sources of information, which have found very little utilisation of such sources [49] and refer to GPs as having “a sort of ‘mental filing cabinet’ of informal information or soft intelligence”[28]. The GPs’ experience with cooperation with various departments or hospitals was very important for the GPs’ choice. GPs’ responses indicated that their experience with specific departments was the most important factor, but many GPs attributed their choice to their experience with a hospital in general rather than the individual department, thereby indicating that they generalised their experience from one or more departments at a hospital to other departments at the hospital as a whole – a kind of ‘halo’-effect.

The strong influence of informal data sources like patients’ previous experience on choice and advice on choice may reflect lack of official information on quality or waiting time or that GPs are suspicious of published data on performance, viewing such data as “spin” [29]. Several respondents commented that use of web-based information was too time-consuming compared to data on paper; their memory of previous referrals, and asking the patient to call one or more hospitals or the county’s patient’s advisors for information.

Some GPs wrote that they intended to use web-based information more in the future. Such statements may reflect expectations that more experience and improved IT will ease their access to the web or lack of experience. When the present study was performed approx. 86% of Danish general practices had access to the internet, and a little less than half of the practices used the access each day [50].

### Implications

Further research is warranted on the interaction between GP and patient in choice of hospital, preferably by direct observation of the referral process followed by interviews with the GP as well as the patient about their views on the referral process including their experience of responsibility for the choice.

The findings in the present study support results from studies of patients’ choice behaviour which indicate that patients and their agents do not act as the autonomous customers assumed in market-resembling models for management of the public sector. When agents act on patients’ behalf they tend to utilise informal sources of information – even when systematically collected and published information on service is available. One implication of the major influence of previous experience with hospital departments may be a tendency to inertia in referral patterns.

### Limitations of the study

The response rate in the present study was 52%, which appears to be quite normal for studies performed in general practice.

The choice of study method meant that we did not observe the process of choice, and only reasons we were aware of beforehand were included in the study, but the questionnaire was validated, and the respondents were offered the opportunity to comment on the reasons and did not refer to reasons not mentioned in the questionnaire.

The respondents could report any number of reasons and we did not ask them to quantify the importance of each reason, because this would complicate the data collection and probably reduce the response rate. We assumed that the cumulative importance of a reason for choice of hospital was proportional to the frequency it was quoted by the GPs, but this may not necessarily be the case: a comparison of two Dutch studies published recently may indicate that frequency of reporting may give results which differ from estimations of importance by way of a choice experiment [25,44].

Respondents did not differ from non-respondents with regard to age, gender or county, but GPs with a stronger than average interest in subjects concerning choice of hospital may be especially likely to participate in the study. Therefore the study may exaggerate the impact of each individual factor on choice of hospital.

Usually studies should be performed prospectively to reduce bias, but in the present study we chose a retrospective design in order not to influence the GPs’ choice behaviour. Our choice of design increased the risk of recall bias, and the GPs may have reported factors which they thought ought to have influenced their choices rather than the decisive factors. For example GPs may have hesitated to quote media reports as an important source of information. GPs probably are very conscious about their use of some sources of information like websites, while the importance of some sources may be underestimated, because their utilisation is more nebulous, like feedback from patients or media reports. Presumably patient characteristics influenced the GPs’ choices but not their willingness to participate. Therefore patient characteristics presumably did not introduce bias in the study.

The study included a large number of statistical tests. Some of the statistically significant associations in univariate analysis may be due to mass significance rather than causality.

The study was performed thirteen years after the introduction of free choice of public hospital within the study area and eleven years after the introduction of free choice of public hospital at the national level. Patients’ awareness of their right to choose was high. Therefore, even though the study was performed at a specific time in the process of introducing free choice of hospital, we find it most likely that studies performed a few years before or after the present study would not have led to results which were very different from those of the present study.

## Conclusions

In an area with free choice of public hospital GPs strongly influenced patients’ choice of hospital by choosing the hospital on their behalf. Short distance to hospital was the factor which most frequently decided the GP’s choice of hospital on behalf of patients. GPs focused on informal sources like feedback from patients and colleagues and their experience with cooperation with the department or hospital, attaching little weight to official information on quality and service (waiting time) at hospitals or departments.

## Competing interests

The authors declare that they have no competing interests.

## Authors’ contributions

Both of the authors conceived and designed the study; HOB developed the questionnaire, collected and analysed the data and wrote the manuscript. LOH assisted in writing the manuscript. Both authors read and approved the final manuscript.

## Pre-publication history

The pre-publication history for this paper can be accessed here:

http://www.biomedcentral.com/1472-6963/12/126/prepub

## Supplementary Material

Additional file 1 **The questionnaire.** The questionnaire used for collecting data for the present study. Click here for file

## References

[B1] Winblad U Ringard A Magnussen J, Vrangbæk K, Saltman RB Meeting rising public expectations: the changing roles of patients and citizens Nordic health care systems. Recent reforms and current policy challenges 2009 Open University Press, Maidenhead

[B2] Le Grand J Motivation, agency, and public policy. Of Knights & Knaves, Pawns & Queens 2003 Oxford University Press, Oxford

[B3] Vrangbæk K Østergren K Patient empowerment and the introduction of hospital choice in Denmark and Norway Health Econ Policy Law 2006 1 371 394 10.1017/S1744133106005032 18634678

[B4] Dixon A Robertson R Appleby J Burge J Devlin N Magee H Patient choice. How patients choose and providers respond 2010 The King’s Fund, London

[B5] Perry G Giving consumers of British public services more choice: what can be learned from recent history? J Soc Policy 2003 32 239 270 10.1017/S0047279402006980

[B6] Thomson S Dixon A Choices in health care: the European experience J Health Serv Res Policy 2006 11 167 171 10.1258/135581906777641703 16824264

[B7] Dowding K John P The value of choice in public policy Public Administration 2009 87 219 233 10.1111/j.1467-9299.2008.01732.x

[B8] Wilmot S A fair range of choice: justifying maximum patient choice in the British National Health Service Health Care Anal 2007 15 59 72 10.1007/s10728-006-0032-6 17628925

[B9] Porter ME Teisberg EO Redefining Health Care. Creating value-based competition on results 2006 Harvard Business School Press, Cambridge

[B10] Appleby J Harrison A Devlin N What is the real cost of more patient choice 2003 King’s Fund, London

[B11] Vrangbæk K The interplay between central and sub-central levels: the development of a systematic standard based programme for governing medical performance in Denmark Health Econ Policy Law 2009 4 305 327 10.1017/S1744133109005040 19467170

[B12] Tai-Seale M Voting with their feet: patient exit and intergroup differences in propensity for switching usual sources of care J Health Polit Policy Law 2004 29 491 514 10.1215/03616878-29-3-491 15328875

[B13] Le Grand J Choice and competition in publicly funded health care Health Econ Policy Law 2009 4 479 488 10.1017/S1744133109990077 19715629

[B14] Hirschman Bernstein AB Gauthier AK Choices in health care: what are they and what are they worth? Med Care Res Rev 1999 56 5 23 10.1177/107755899773743846 10354677

[B15] Bernstein AB Gauthier AK Choices in health care: what are they and what are they worth? Med Care Res Rev 1999 56 5 23 10.1177/107755899773743846 10354677

[B16] Martinsen DS Vrangbæk K The Europeanization of health care governance: implementing the market imperatives of Europe Public Administration 2008 86 169 184 10.1111/j.1467-9299.2007.00702.x

[B17] Fotaki M Roland R Boyd A McDonald R Scheaff R Smith L What benefits will choice bring to patients? Literature review and assessment of implications J Health Serv Res Policy 2008 13 178 184 10.1258/jhsrp.2008.007163 18573768

[B18] Marshall MN Shekelle PG Leatherman S Brook RH The public release of performance data: what do we expect to gain? A review of the evidence JAMA 2000 283 1866 1874 10.1001/jama.283.14.1866 10770149

[B19] Akerlof GA The market for “lemons”. Quality uncertainty and the market mechanism Q J Econ 1970 84 488 500 10.2307/1879431

[B20] Arrow KJ Uncertainty and the welfare economics of health care Am Econ Rev 1963 53 941 973

[B21] Loomes G Sugden R Regret theory: an alternative theory of rational choice under uncertainty Econ J 1982 92 805 824 10.2307/2232669

[B22] Bell D Regret in decision making under uncertainty Oper Res 1982 30 961 981 10.1287/opre.30.5.961

[B23] Barnett J Ogden J Daniells E The value of choice: a qualitative study Br J Gen Pract 2008 58 609 613 10.3399/bjgp08X330717 18801277 PMC2529197

[B24] Rosen R Curry Florin D Public views on choices in health and health care 2005 King’s Fund, London

[B25] Djis-Elsinga J Otten W Versluijs MM Smeets HJ Kievit J Vree R van der Made WJ Marang-van de Mheen P Choosing a hospital for surgery: the importance of information on quality of care Med Decis Making 2010 30 544 555 10.1177/0272989X09357474 20110514

[B26] Østerbye T Gut R Blæsbjerg C Freil M Patienters oplevelser på landets sygehuse 2004 [in Danish: Patients’ Experience at Danish Hospitals 2004] 2005 Enheden for Brugerundersøgelser, Glostrup

[B27] Department of Health Report on the National Patient Choice Survey, February 2010 England [online]. Available at http://www.dh.gov.uk/prod_consum_dh/groups/dh_digitalassets/documents/digitalasset/dh_117096.pdf [homepage on the Internet] [cited on 1 April 2012]

[B28] Burge P Devlin N Appleby J Gallo F Nason E Ling T Understanding patients’ choices at the point of referral 2006 RAND Europe, Cambridge

[B29] Rosen R Florin D Hutt R The anatomy of GP referral decisions. A qualitative study of GPs’ views on their role in supporting patient choice 2007 King’s Fund, London

[B30] Wiinblad U Do physicians care about patient choice? Soc Sci Med 2008 67 1502 1511 10.1016/j.socscimed.2008.07.016 18786753

[B31] Strandberg-Larsen M Nielsen MB Vallgårda S Krasnik A Vrangbæk K Mossialos E Denmark: Health system review Health Syst Transit 2007 9 6 1 164

[B32] Vrangbæk K Markedsorientering i sygehussektoren [in Danish: Market orientation in the hospital sector]. PhD-thesis 1999 Institute of Political Science, University of Copenhagen, Copenhagen

[B33] Mahon A Whitehouse C Wilkin D Nocon A Factors that influence general practitioners’ choice of hospital when referring patients for elective surgery Br J Gen Pract 1993 43 272 276 8398242 PMC1372452

[B34] Odell A A study of patient referrals Publ Hlth Lond 1983 97 109 114 10.1016/S0033-3506(83)80007-9 6856728

[B35] Kennedy F McConnell General practitioner referral patterns J Public Health Med 1993 15 83 87 8471305 10.1093/oxfordjournals.pubmed.a042824

[B36] Ringard A Rico A Introducing patient choice of hospital in National Health Systems – A comparison of the UK and Norway. Working Paper 2006 Health Organization Research Norway (HORN), Oslo

[B37] Edwards P Roberts I Clarke M DiGuiseppi C Pratap S Wentz R Kwan I Increasing response rates to postal questionnaires: systematic review BMJ 2002 324 1183 1191 10.1136/bmj.324.7347.1183 12016181 PMC111107

[B38] , Lov om et videnskabsetisk komitésystem og behandling af biomedicinske forskningsprojekter [in Danish: Act on a Biomedical Ethics Committee System and the Processing of Biomedical Research Projects]. Available in English from: http://www.cvk.sum.dk/English/actonabiomedicalresearch.aspx [homepage on the Internet] [cited on 1 April 2012]

[B39] Ringard Å Why do general practitioners abandon the local hospital? An analysis of referral decisions related to elective treatment Scand J Public Health 2010 38 597 604 10.1177/1403494810371019 20501548

[B40] Dixon A Robertson R Bal R The experience of implementing choice at point of referral: a comparison of the Netherlands and England Health Econ Policy Law 2010 5 295 317 10.1017/S1744133110000058 20462469

[B41] Mol A The logic of care. Health and the problem of patient choice 2008 Routledge, Abingdon

[B42] de Mheen PJ Marang-van Dijs-Elsinga J Otten W Versluijs M Smeets HJ Vree R van der Made WJ Kievit J The relative importance of quality of care information when choosing a hospital for surgical treatment: a hospital choice experiment Med Decis Making 2011 31 816 10.1177/0272989X11386799 22067430

[B43] Adams EK Wright KE Hospital choice of Medicare beneficiaries in a rural market: why not the closest? J Rural Health 1991 7 134 152 10.1111/j.1748-0361.1991.tb00715.x 10116774

[B44] McArdle PJ Whitnall M The referral practice of general medical practitioners to the surgical specialties: implications for the future Br J Oral Maxillofac Surg 1996 34 394 399 10.1016/S0266-4356(96)90094-7 8909729

[B45] Morrell DC Roland MO Analysis of referral behaviour: responses to simulated case histories may not reflect real clinical behaviour Br J Gen Pract 1990 40 182 185 2114132 PMC1371274

[B46] Conner-Spady B Sanmartin C Johnston G McGurran J Kehler M Noseworthy T Willingness of patients to change surgeons for a shorter waiting time for joint arthroplasty CMAJ 2008 179 327 332 10.1503/cmaj.071659 18695180 PMC2492973

[B47] Dunn E Black C Alonso J Nørregaard JC Anderson GF Patients’ acceptance of waiting time for cataract surgery: what makes a wait too long? Soc Sci Med 1997 44 1603 1610 10.1016/S0277-9536(96)00251-1 9178406

[B48] De Groot IB Otten W Smeets HJ Marang-van de Mheen PJ Is the impact of hospital performance data greater in patients who have compared hospitals? BMC Health Serv Res 2011 11 214 10.1186/1472-6963-11-214 21906293 PMC3203258

[B49] Burge P Devlin N Appleby J Rohr C Grant J Do patients always prefer quicker treatment?: a discrete choice analysis of patients’ stated preferences in the London Choice Project Appl Health Econ Health Policy 2004 3 183 194 10.2165/00148365-200403040-00002 15901193

[B50] Praksistælling 2003 [in Danish: ” General Practices in 2003”] 2003 The Danish General Practitioners Organisation, Copenhagen

