# Supplementary material for: Which factors decided general practitioners’ choice of hospital on behalf of their patients in an area with free choice of public hospital? A questionnaire study
Source: BMC Health Serv Res. 2012 May 25;12:126. doi: 10.1186/1472-6963-12-126 (PMC3407516; doi:10.1186/1472-6963-12-126)
Supplement: Additional file 1 — The questionnaire. The questionnaire used for collecting data for the present study. [file 1472-6963-12-126-S1.doc]

**Additional file 1: Extract from the questionnaire:**

**Study of general practitioners’ advice to patients on choice of hospital, The Three-County-Area, 2003.**

**1 Information about the individual participants**

Gender: Male □ Female □

Year of birth: 19____

**The present study solely concerns the latest three elective patients you referred to a clinical hospital department or a clinical out-patient-clinic**

**2 Key data about the latest three patients:**

**Patient 1:**

Year of birth: 19____

Gender: Male □ Female □

Specialty: __________________________

**Patient 2:**

Year of birth: 19____

Gender: Male □ Female □

Specialty: __________________________

**Patient 3:**

Year of birth: 19____

Gender: Male □ Female □

Specialty: __________________________

**3 Who chose the hospital on be**half of the latest three patients?

|  | Patient 1 | Patient 2 | Patient 3 |
| --- | --- | --- | --- |
| I did |  |  |  |
| The patient did |  |  |  |
| The patient’s relatives did |  |  |  |

Comments: ___________________________________________________________

_____________________________________________________________________

**4** Which factors were decisive for the choice of hospital for the three patients?

|  | Pat. 1 | Pat. 2 | Pat. 3 |
| --- | --- | --- | --- |
| The department was the closest one to the patient’s home |  |  |  |
| The patient had been treated at the hospital before |  |  |  |
| The patient had been treated at the department before |  |  |  |
| The waiting time was shorter than at other departments |  |  |  |
| Comments from patients which I have referred to the hospital |  |  |  |
| Comments from patients which I have referred to the department |  |  |  |
| My experience as a trainee at the hospital |  |  |  |
| My experience as a trainee at the department |  |  |  |
| The hospital takes the my referrals seriously |  |  |  |
| The department takes my referrals seriously |  |  |  |
| The hospital provides detailed clinical reports |  |  |  |
| The department provides detailed clinical reports |  |  |  |
| The hospital sends clinical reports soon after discharge |  |  |  |
| The department sends clinical reports soon after discharge |  |  |  |
| Excellent cooperation between GP and department |  |  |  |
| I cannot remember |  |  |  |

Comments (and other reasons): ___________________________________________

_____________________________________________________________________

_____________________________________________________________________

**5 What are your most important sources of information about quality at hospital departments?**

Information from other GPs about the hospital □

Information from other GPs about the department □

The hospital’s description of its quality standards □

The department’s description of its quality standards □

Official information from the hospital □

Official information from the department □

Information meetings at hospital departments □

Experience as a trainee at the department □

Experience as a trainee at the hospital □

My experience with acquaintances who work at the department or the hospital □

Comparisons of clinical reports from different departments □

Information from patients discharged from the hospital □

Information from patients discharged from the department □

Media reports about the hospital □

Media reports about the department □

Comments (and other sources): ___________________________________________

_____________________________________________________________________

_____________________________________________________________________

_____________________________________________________________________

**6 How often do you use different sources of information about waiting time?**

Routinely Often Rarely Not at all

Confirmations of referrals □ □ □ □

and clinical reports

Calls to the departments □ □ □ □

The counties’ prognoses (paper) □ □ □ □

The counties prognoses (the web) □ □ □ □

[www.venteinfo.dk](http://www.venteinfo.dk/) by the Ministry □ □ □ □

of the Interior and Health

[www.sundhed.dk](http://www.sundhed.dk/) □ □ □ □

Comments (and other sources): ___________________________________________

_____________________________________________________________________

_____________________________________________________________________

_____________________________________________________________________
